# Transcriptional profiling of bovine intervertebral disc cells: implications for identification of normal and degenerate human intervertebral disc cell phenotypes

**DOI:** 10.1186/ar2929

**Published:** 2010-02-11

**Authors:** Ben M Minogue, Stephen M Richardson, Leo AH Zeef, Anthony J Freemont, Judith A Hoyland

**Affiliations:** 1 Tissue Injury and Repair, School of Biomedicine, Faculty of Medical and Human Sciences, University of Manchester, Oxford Road, Manchester, M13 9PT, UK; 2 Faculty of Life Sciences, Michael Smith Building, University of Manchester, Oxford Road, Manchester, M13 9PT, UK

## Abstract

**Introduction:**

Nucleus pulposus (NP) cells have a phenotype similar to articular cartilage (AC) cells. However, the matrix of the NP is clearly different to that of AC suggesting that specific cell phenotypes exist. The aim of this study was to identify novel genes that could be used to distinguish bovine NP cells from AC and annulus fibrosus (AF) cells, and to further determine their expression in normal and degenerate human intervertebral disc (IVD) cells.

**Methods:**

Microarrays were conducted on bovine AC, AF and NP cells, using Affymetrix Genechip^® ^Bovine Genome Arrays. Differential expression levels for a number of genes were confirmed by quantitative real time polymerase chain reaction (qRT-PCR) on bovine, AC, AF and NP cells, as well as separated bovine NP and notochordal (NC) cells. Expression of these novel markers were further tested on normal human AC, AF and NP cells, and degenerate AF and NP cells.

**Results:**

Microarray comparisons between NP/AC&AF and NP/AC identified 34 NP-specific and 49 IVD-specific genes respectively that were differentially expressed ≥100 fold. A subset of these were verified by qRT-PCR and shown to be expressed in bovine NC cells. Eleven genes (SNAP25, KRT8, KRT18, KRT19, CDH2, IBSP, VCAN, TNMD, BASP1, FOXF1 & FBLN1) were also differentially expressed in normal human NP cells, although to a lesser degree. Four genes (SNAP25, KRT8, KRT18 and CDH2) were significantly decreased in degenerate human NP cells, while three genes (VCAN, TNMD and BASP1) were significantly increased in degenerate human AF cells. The IVD negative marker FBLN1 was significantly increased in both degenerate human NP and AF cells.

**Conclusions:**

This study has identified a number of novel genes that characterise the bovine and human NP and IVD transcriptional profiles, and allows for discrimination between AC, AF and NP cells. Furthermore, the similarity in expression profiles of the separated NP and NC cell populations suggests that these two cell types may be derived from a common lineage. Although interspecies variation, together with changes with IVD degeneration were noted, use of this gene expression signature will benefit tissue engineering studies where defining the NP phenotype is paramount.

## Introduction

Low back pain (LBP) is the leading cause of disability and sick leave in the UK and it has been estimated that more than 80% of the population will report LBP at some point during their lifetime [1]. Each year as a result of sick leave, disability benefits and medical and insurance costs, LBP costs the British economy alone over £12 billion [2]. One of the main causes of LBP is thought to be degeneration of the intervertebral disc (IVD) [3]. However, current treatments for IVD degeneration and LBP are aimed at relieving symptoms rather than being curative and offer little hope of restoring the IVD to its original function. Consequently, there is an urgent need for a more effective treatment of IVD degeneration. Recent advances in tissue engineering and IVD biology offer exciting potential therapies for repairing the IVD, in particular, via the introduction of differentiated mesenchymal stem cells (MSCs) into the degenerate nucleus pulposus (NP). In recent years, several *in vitro *and *in vivo *studies have demonstrated that MSCs are capable of differentiation into chondrogenic cells, similar to those found in the NP of the disc [4-9]. However, in order for any tissue engineering strategy aimed at repairing the degenerate NP to be successful, it is crucial that the definitive molecular phenotype of NP cells is elucidated.

Each IVD is comprised of three morphologically distinct regions; the cartilaginous end plates (CEP), the ligamentous annulus fibrosus (AF) and the gelatinous NP. Cells of the AF and NP have previously been described as chondrocyte-like cells [10] but markedly differ from each other and articular chondrocytes. AF cells are elongated and fibroblastic in appearance, but retain expression of chondrocyte marker genes, such as type II collagen (COL2A1) and aggrecan (ACAN). NP cells demonstrate a classic rounded chondrocyte-like morphology and express a number of chondrocyte marker genes [11], although their origin and full molecular phenotype are not clearly understood. Complicating this further is the presence of a second cell population within the NP. During development the perichordal disc, forerunner of the IVD and endplates, forms by segmentation of the mesenchymal column that surrounds the developing notochord (NC). The notochordal segments expand in cell number and mucoid extracellular matrix (ECM) to form the notochordal NP [12,13]. In humans, this population of NC cells present during development is gradually replaced by a population of smaller, spherical NP cells [14]. However, in many animal species these larger, 'physaliferous' notochordal cells persist throughout the life time of the animal. In species where NC cells persist, the NP ECM demonstrates a higher level of hydration than in adult human NP and there is no evidence of IVD degeneration, suggesting that NC cells play an important role in regulating disc cell function and ECM synthesis [15-17].

Although NP cells have a phenotype similar to articular chondrocytes [11], the ECM in which they reside shows distinct differences in proteoglycan (PG) and collagen contents. In the NP, the PG:collagen ratio is approximately 27:1, whereas in articular cartilage (AC) is reported to be 2:1 resulting in a less fibrous, more highly hydrated tissue in the NP [18]. Such ECM differences imply that for correct functioning of an engineered IVD it is essential for the implanted cells to possess the correct phenotype. Importantly, for stem cells-based tissue regeneration it is therefore not sufficient to rely on pre-existing chondrocyte marker genes to define an NP phenotype.

Surprisingly, there have been few attempts to characterise the differences in the transcriptional or protein profiles of IVD cells and AC cells. Hypoxia inducible factor 1 isoforms (HIF1A and HIF1B), glucose transporter type 1 (GLUT-1), matrix metalloproteinase 2 (MMP-2) and vascular endothelial growth factor (VEGF) have been postulated as NP cell marker genes, with their presence identifying an adaptation of the cells to the unique environment of the IVD [19-21]. This suggests that the challenging environment of the IVD contributes to defining the cells that occupy the NP. Fujita and colleagues [22] used rat NP to identify cell surface markers that were specific to NP cells and identified CD24, a glycosylphosphatidylinositol anchor protein, as being highly expressed in NP cells in a tissue specific manner. However, no data were presented for differences between human NP and AC cells, so it remains unclear whether CD24 can indeed be used for distinguishing human NP cells.

More recently studies have utilised microarrays in rat and canine tissues to compare phenotypes of IVD cells and articular chondrocytes and have reported a number of genes that are differentially expressed in NP, AF and AC cells. Lee and colleagues [23] identified 63 genes between rat NP and AF cells and 41 genes between NP and AC cells with at least five-fold differences in expression. A handful of these potential marker genes were further characterised and although the authors observed no clear on/off markers, keratin 19 and glypican 3 (GPC3) encouragingly stained immunopositive in the NP of discs from young rats but were negative in AF and AC cells. When the same group studied canine NP and AF cells, they identified 45 genes that were more highly expressed in NP than AF cells [24]. Differential expression of five of these genes was then confirmed using quantitative real-time PCR (qRT-PCR) on NP, AF and AC cells, which demonstrated that α-2-macroglobulin (A2M), cytokeratin-18 (KRT18) and neural cell adhesion molecule (NCAM1) were enhanced in NP compared with AC cells. No difference was noted in desmocollin 2 (DSC2) or annexin A4 (ANXA4) between NP and AF or NP, AF and AC cells, respectively. Furthermore, when five of the differentially expressed genes identified in the earlier rat arrays (cartilage oligomeric matrix protein (COMP), GPC3, matrix Gla protein (MGP), pleiotrophin (PTN) and vimentin (VIM)) were studied in the canine tissues, only 2 (COMP and MGP - markers of AC cells rather than NP cells) demonstrated a similar pattern in the canine IVD. This suggests that interspecies variations in gene expression exists, although the fact that rat discs retain a high notochordal cell population, whereas the beagle dogs used in their study are non-notochordal may explain the differences observed. Crucially, such data highlight the importance of identifying both cell type-specific and species-specific genes when defining the IVD cell phenotype.

The aim of this study was to utilise Affymetrix microarray technology to identify novel bovine marker genes that could be used to discriminate the transcriptional profile of chondrocyte-like NP cells from AC cells. Bovine caudal tissues were chosen because they are frequently used as models for testing regenerative medicine therapies due to their similarity in size and physico-chemical environment to human lumbar discs [25]. Differential expression of a number of genes identified by microarray was validated using qRT-PCR on bovine NP, AF and AC cells and then examined in NC cells isolated from immature bovine caudal discs. These validated, differentially expressed genes were then further analysed in human IVD and AC cells to assess their feasibility as markers of NP cells across species. Finally, any change in these marker genes with IVD degeneration was assessed using qRT-PCR on both non-degenerate and degenerate human IVD cells.

## Materials and methods

### Tissue and sample preparation for cDNA microarray

Bovine IVD tissue was obtained from bovine tails of young adult animals (18 to 36 months old) purchased from a local slaughterhouse. The discs were excised and macroscopically dissected into AF and NP tissues taking care to remove any transition zone. AC was also isolated from the stifle joints of the same animals and the three tissue types from each individual were cut into 2 to 3 mm^3 ^fragments. Each sample was enzymatically digested in serum-free media containing 0.5% pronase (Merck Chemicals Ltd, Nottingham, UK) for one hour and transferred to serum-free media containing 0.5% collagenase type II (Invitrogen, Paisley, UK) and 0.1% hyaluronidase (Sigma, Poole, UK) for two to three hours on an orbital shaker at 37°C. Supernatant was passed through a 40 μm filter to remove tissue debris. Cells were then collected by centrifugation at 500 *G *for five minutes and the cell pellet lysed in Trizol^® ^reagent (Invitrogen, Paisley, UK). RNA was extracted from the recovered cells with the addition of high salt precipitation solution (HSPS) as recommended by the manufacturer, quantified using a Nanodrop ND-1000 spectrophotometer (Nanodrop Technologies, Wilmington, DE, USA) and quality checked using the RNA 6000 Nano Assay analysed on the Agilent 2100 Bioanalyzer (Agilent Technologies, Stockport, UK). Only high-quality RNA with an RNA integrity number (RIN) of at least seven was used for the arrays. To minimise the effect of biological variation on differential expression, RNA was pooled from five animals for each cell type and hybridisations for each cell type were performed in triplicate (15 animals in total).

### cDNA microarrays

Microarray experiments were performed using the Genechip^® ^Bovine genome arrays (Affymetrix, High Wycombe, UK). For each hybridisation, 15 μg of total RNA was used to prepare first-strand cDNA using an oligo (dT)-T7 primer. Following second-strand synthesis, biotinylated cRNA targets were generated using an Enzo BioArray high yield RNA transcript-labeling kit (Affymetrix, High Wycombe, UK) by *in vitro *transcription with biotinylated UTP and CTP. Technical quality control was performed with dChip [26]. Principal component analysis (PCA) was performed using Partek software, version 6.0 (Partek Inc., St. Charles, MO, USA) to demonstrate overall variance in gene expression between the three cell types [27] and gene expression and statistical analysis was performed using PUMA software (The University of Manchester, Manchester, UK) [28]. The false discovery rate estimation was obtained by performing a parametric analysis of variance (ANOVA) and false discovery correction by the qvalue method (Princeton University, Princeton, NJ, USA) [29]. The microarray data was submitted in MIAME (Minimum Information About a Microarray Experiment) compliant format to the ArrayExpress database ([30], accession number [ArrayExpress:E-MEXP-2291]).

### Sample preparation for quantitative real time PCR

#### Bovine Samples

qRT-PCR with gene-specific primers was performed in triplicate on cDNA derived from each of the three different tissues, NP, AF and AC, from five individual animals as described above. Additionally, notochordal cells were separated from NP cells using enzymatic digestion as described above and the cells in the supernatant separated further by using a sequential cell sieving method [16]. Large 'notochordal' cells retained in the 15 μm and 10 μm sieves (CellMicroSieves, BioDesign Inc., New York, NY, USA) were washed from the membrane, collected by centrifugation at 500 *G *for five minutes and the cell pellet lysed in Trizol^®^. Cells in the supernatant that had passed through the 8 μm filter were also collected by centrifugation at 500 *G *for five minutes and the cell pellet lysed in Trizol^®^. RNA for both cell types was extracted as described above. Of each RNA sample, 2 μg was treated with DNAse I (Invitrogen, Paisley, UK) to remove contaminating genomic DNA and reverse transcribed into cDNA using the high capacity cDNA reverse transcription kit (Applied Biosystems, Warrington, UK) according to manufacturer's instructions. cDNA samples were diluted to 5 ng/μl using molecular grade water prior to use.

#### Human samples

Human IVD tissue was obtained during post-mortem examination with informed consent from relatives and local ethical committee approval (North West Research Ethics committee). Samples were further dissected for histological grading, using a published histological 12-point scale [11], and for enzymatic digestion into NP and outer AF regions, ensuring that transition zone tissue was removed. Five non-degenerate (histological grade 1 to 3: ages 45 to 60 years; mean age 52 years) and five moderately degenerate (grades 6 to 8; ages 49 to 57 years; mean age 51 years) discs were used. Human AC was obtained, with informed consent and local ethical approval (South Manchester Research Ethics committee), during total knee arthroplasty from patients with osteoarthritis. Five AC samples (ages 50 to 60 years; mean age 56 years) from the medial and lateral compartments (femoral condyles and tibial plateaus) with a 'normal' macroscopic appearance were harvested and full thickness sections excluding subchondral bone were fixed in formalin and processed to paraffin wax for histology. Sections were stained with H&E and safranin-O staining and graded using an 11 point scoring system adapted from Mankin and colleagues [31] as previously described [32]. Only samples from areas of cartilage graded as histologically 'normal' were used. Cells from each of the AC, AF and NP tissues were enzymatically released and the RNA recovered and prepared for qRT-PCR as described for bovine samples.

### Quantitative real-time PCR

qRT-PCR was carried out on cDNA samples using the SYBR^® ^Green method. Gene-specific primers (Tables 1 and 2) were designed using the Primer Express 2 software (Applied BioSystems, Warrington, UK), optimised to ensure specificity and tested against either a bovine genomic DNA standard curve or human universal reference total RNA (Takara Bio Europe/Clontech, Saint-Germain-en-Laye, France) standard curve, to ensure optimal efficiency. Reactions were conducted on an ABI Prism 7000 sequence detection system (Applied BioSystems, Foster City, CA, USA) in triplicate in 96-well plates in a final volume of 20 μl under standard conditions. Reaction mixes contained 10 μl of two times SYBR Green mastermix (Applied BioSystems, Warrington, UK), 1 μl (6 μM) forward primer, 1 μl (6 μM) reverse primer, 6 μl water and 2 μl (5 ng/μl) cDNA. The 2^-ΔCt ^and 2^-ΔΔCt ^methods were used to calculate relative expression of each target gene as described previously [33,34]. For the 2^-ΔCt ^method, mean Ct values of target genes in each sample was normalised to the housekeeping gene values. The 2^-ΔΔCt ^method was used to analyse changes in gene expression between normal and degenerate human tissues, where the ΔCt value in the degenerate tissues was normalised to the ΔCt value in the non-degenerate samples to give the ΔΔCt value. Statistical analysis was performed with GraphPad InStat software (GraphPad Software, Inc. La Jolla, CA, USA) using the Mann-Whitney U-test and significance was defined as *P *< 0.05.

**Table 1:** Bovine oligonucleotide primers

Gene name	Gene symbol	NCBI ref. seq.	Forward primer	Reverse primer
Glyceraldehyde-3-phosphate dehydrogenase	GAPDH	NM_001034034.1	TGCCGCCTGGAGAAACC	CGCCTGCTTCACCACCTT
Aggrecan	ACAN	NM_173981.2	GGGAGGAGACGACTGCAATC	CCCATTCCGTCTTGTTTTCTG
Versican	VCAN	NM_181035.2	GCTGCATGCCGCCTATG	TCCGTAGGTCCGGACTCCTT
Collagen, type II, alpha 1	COL2A1	NM_001001135.2	CGGGCTGAGGGCAACA	CGTGCAGCCATCCTTCAGA
Synaptosomal associated protein 25	SNAP25	NM_001076246.1	GGCTTCATCCGCAGGGTAA	GCTCCAGGTTTTCATCCATTTC
Keratin 8	KRT8	NM_001033610.1	ACCAGGAGCTCATGAATGTCAA	TCGCCCTCCAGCAGCTT
Keratin 18	KRT18	XM_582930.4	TTGAGCTGCTCCATCTGCAT	AAGGCCAGCTTGGAGAACAG
Keratin 19	KRT19	XM_875997.3	CGGTGCCACCATTGAGAACT	CAAACTTGGTGCGGAAGTCA
N-Cadherin	CDH2	XM_001250829.2	GCCATCAAGCCAGTTGGAA	TGCAGATCGAACCGGGTACT
Sclerostin domain containing 1	SOSTDC1	NM_001046265.1	GTTCAAGTAGGCTGCCGAGAA	GCACTGGCCGTCTGAGATG
Integrin-binding sialoprotein	IBSP	NM_174084.2	GACAGCTATGATGGTCAAGATTACTACA	TGGGTGAACTCATCCCAGTCT
Tenomodulin	TNMD	NM_001099948.1	TCTGGCGTGACGGGTCTT	AAAAAAGGCATTGAACAAAACGA
Brain attached signalling protein 1	BASP1	NM_174780.3	TTGTGGATGAATGCCAACTTTC	AAAAATGGAGTATTGGCATCAAGAT
TNF, alpha-induced protein 6	TNFAIP6	NM_001007813.1	AAGCAGCAGGCGTCTACCA	CACACCGCCTTCGCTTCT
Forkhead box F1	FOXF1	XM_603148.4	TCCCTCCCCACCTCAGAAGT	TGGCTTCAGAAATGCAAGTTACTC
Forkhead box F2	FOXF2	CK941878.1	TGCGTGGTAAGTTTTCACCATCT	CCCCCGGTGAGGTAATGC
Aquaporin 1	AQP1	NM_174702.3	ACCAGGAGGCCCTTAATGGT	CTGTTAAATGGACTAGAAGCGAAATG
Fibulin 1	FBLN1	NM_001098029.1	GCAGCGCAGCCAAGTCAT	AGATATGTCTGGGTGCTACAAACG
T, brachyury homolog (mouse)	T	XM_864890.2	ACTTCGTGGCGGCTGACA	GCACCCACTCCCCATTCA

Bovine oligonucleotide primers used for quantitative real-time polymerase chain reaction (qRT-PCR).

**Table 2:** Human oligonucleotide primers

Gene name	Gene symbol	NCBI ref. seq.	Forward primer	Reverse primer
Actin, beta	ACTB	NM_001101.3	CGAGAAGATGACCCAGATCATG	ACAGCCTGGATAGCAACGTACA
Aggrecan	ACAN	NM_001135.2	TCTACCGCTGCGAGGTGAT	TGTAATGGAACACGATGCCTTT
Versican	VCAN	NM_004385.4	GCCTTTCCTATCACCTCGAGAA	CACGGCAACCCAAAATGACT
Collagen, type II, alpha 1	COL2A1	NM_001844.4	GGAAGAGTGGAGACTACTGGATTGAC	TCCATGTTGCAGAAAACCTTCA
Synaptosomal associated protein 25	SNAP25	NM_003081.2	CAATGAGCTGGAGGAGATGCA	TGCTTTCCAGCGACTCATCA
Keratin 8	KRT8	NM_002273.3	CACATCTGTGGTGCTGTCCAT	GCCTTGACCTCAGCAATGATG
Keratin 18	KRT18	NM_000224.2	GCCTACAAGCCCAGATTGC	GGCGAGGTCCTGAGATTTGG
Keratin 19	KRT19	NM_002276.4	CGCAGGGTGCTGGATGAG	AGGTAGGCCAGCTCTTCCTT
N-Cadherin	CDH2	NM_001792.3	AGCCTGGAACGCAGTGTAC	GCGAACCGTCCAGTAGGAT
Integrin-binding sialoprotein	IBSP	NM_004967.3	CCAGAGGAAGCAATCAC	GCACAGGCCATTCCCAA
Tenomodulin	TNMD	NM_022144.2	CAGTGGGTGGTCCCTCAAG	GTCATTTATTGGAAGTTCTTCCTCACTTG
Brain attached signalling protein 1	BASP1	NM_006317.3	GCGGAGCCCGAGAAGAC	GGCCTCAGCAGCTTTGG
Forkhead box F1	FOXF1	NM_001451.2	AAGCCGCCCTATTCCTACATC	GCGCTTGGTGGGTGAACT
Fibulin 1	FBLN1	NM_006487.2	CCTTCGAGTGCCCTGAGAACTA	ACCGATGGCCTCATGCA
T, brachyury homolog (mouse)	T	NM_003181.2	CAATGAGATGATCGTGACCAAGA	GCCAGACACGTTCACCTTCA

Human oligonucleotide primers used for quantitative real-time polymerase chain reaction (qRT-PCR).

## Results

### Principal component analysis

**Figure 1:**
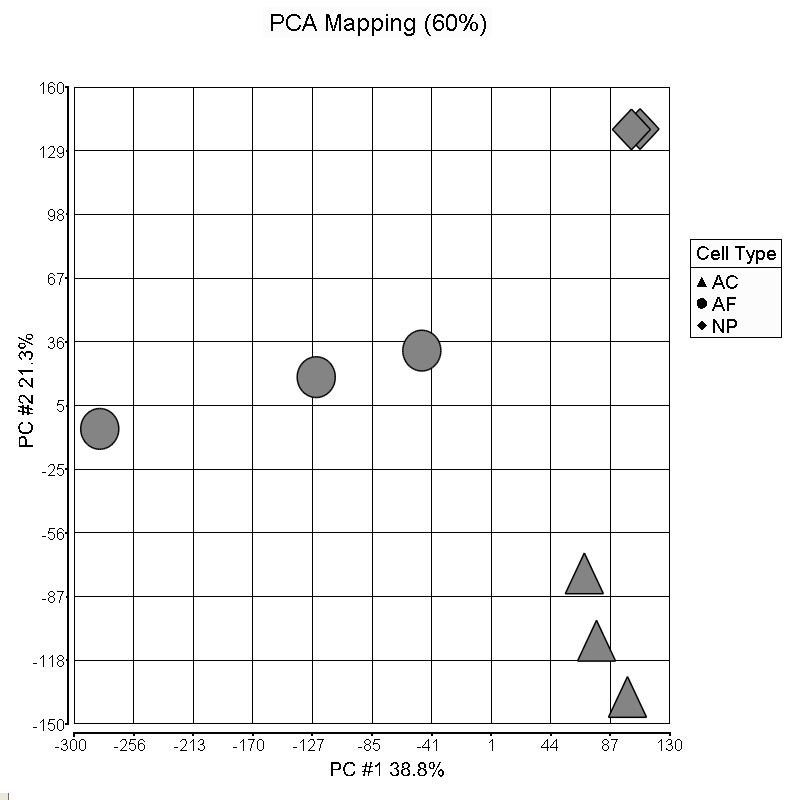
**Principal component analysis (PCA) of microarray data set**. Overall variation between the three cell types articular cartilage (AC; triangles), annulus fibrosus (AF; circles) and nucleus pulposus (NP; diamonds), where each spot represents an individual array, can be seen by the clustering within each cell type and the separation between the different cell types.

The results of the PCA of the microarray data showed that the three replicates for each cell type clustered closely together validating our ability to separate the cell types by dissection and revealing distinct expression profiles for the AC, AF and NP cell types (Figure 1). PCA mapping showed that 60% of the overall variance in the microarray dataset is described by the first two principal components. On the first principal component (which describes 38.8% of the overall variance) AF cells were shown to have a different expression profile to AC and NP cells suggesting that AC and NP cells are more similar to each other than the AF cell type. The second principal component (which describes 21.3% of the overall variance) separates AC from NP cells indicating that clear differences also exist between these two cell types, although they are less dramatic than with the AF cell type.

### Microarray identification of cell-specific 'marker' genes

To assess the ability of the microarray to correctly identify gene expression within the tissues, expression of typical chondrocyte markers was determined (Table 3). The results demonstrated that pre-existing marker genes generally showed the expected pattern of expression, that is high expression of ACAN in NP and AC, high expression of type I collagen in AF compared with NP and AC, and high expression of versican (VCAN) in both NP and AF compared with AC.

**Table 3:** Chondrogenic markers

Gene description	Gene symbol	AC mean	AF mean	NP mean	Fold change (NP/AC)	Fold change (NP/AF)	Fold change (NP/AC&AF)
collagen, type I, alpha 1	COL1A1	21.11	2716.16	260.02	**12.32**	**-10.45**	**-6.77**
collagen, type I, alpha 2	COL1A2	1009.68	15436.11	1330.31	**1.32**	**-11.60**	**-6.51**
collagen, type II, alpha 1	COL2A1	13485.23	13260.79	4436.71	**-3.04**	**-2.99**	**-3.12**
SRY-box 9	SOX9	49.26	8.47	60.38	**1.23**	**7.13**	**1.86**
aggrecan	ACAN	2852.28	168.13	2467.99	**-1.16**	**14.68**	**1.09**
versican	VCAN	21.36	1109.99	1864.91	**87.30**	**1.68**	**2.59**

Bovine microarray data for typical chondrogenic markers. Mean fluorescence intensity expression is shown for each cell type (AC, AF and NP) along with the calculated fold change values. AC, articular cartilage; AF, annulus fibrosus; NP, nucleus pulposus.

Additionally, a number of genes previously described as being phenotypic NP markers were assessed to determine their expression in bovine cells (Table 4). All genes previously suggested as phenotypic NP markers were expressed by bovine IVD and AC cells. However, none of these exhibited high differential expression, with the exception of KRT19 identified from the rat microarray study [23], which showed approximately a 20-fold increase in NP cells compared with AC and AF cells, and KRT18 and NCAM1 identified from the canine microarray study [24], which demonstrated 1860-fold and 10-fold higher expression in the NP cells than AF or AC cells, respectively.

**Table 4:** Previously identified marker

Gene description	Gene symbol	AC mean	AF mean	NP mean	Fold change (NP/AC)	Fold change (NP/AF)	Fold change (NP/AC&AF)
matrix metallopeptidase 2	MMP2	200.20	155.41	121.53	**-1.65**	**-1.28**	**-1.56**
solute carrier family 2, member 1	SLC2A1	1200.57	1211.32	1281.16	**1.07**	**1.06**	**1.04**
hypoxia-inducible factor 1, alpha	HIF1A	3985.62	7783.65	2552.07	**-1.56**	**-3.05**	**-2.50**
vascular endothelial growth factor	VEGF	1874.33	5661.88	2233.09	**1.19**	**-2.54**	**-1.86**
CD24 molecule	CD24	521.84	44.59	86.75	**-6.02**	**1.95**	**-3.42**

keratin 19	KRT19	0.91	0.65	32.59	**35.81**	**50.47**	**19.88**
glypican 3	GPC3	4.24	37.63	13.28	**3.13**	**-2.83**	**-1.78**
annexin A3	ANXA3	7.64	63.66	8.26	**1.08**	**-7.71**	**-7.44**
pleiotrophin	PTN	208.08	54.11	1.10	**-189.18**	**-49.19**	**-152.58**
vimentin	VIM	4625.18	4749.33	3508.86	**-1.32**	**-1.35**	**-1.35**
cartilage oligomeric matrix protein	COMP	6053.50	2986.89	2786.96	**-2.17**	**-1.07**	**-1.78**
matrix Gla protein	MGP	8480.52	7742.10	2865.30	**-2.96**	**-2.70**	**-3.21**

alpha-2-macroglobulin	A2M	9.49	26.62	11.37	**1.20**	**-2.34**	**-1.77**
annexin A4	ANXA4	1223.21	2318.40	1273.29	**1.04**	**-1.82**	**-1.55**
desmocollin 2	DSC2	1.88	140.72	68.18	**36.30**	**-2.06**	**-1.22**
keratin 18	KRT18	0.01	0.04	94.10	**15990.45**	**2647.60**	**1860.77**
neural cell adhesion molecule 1	NCAM1	0.76	0.94	12.64	**16.55**	**13.43**	**10.06**

Bovine microarray data for previously identified markers. Mean fluorescence intensity expression is shown for each cell type (AC, AF and NP) along with the calculated fold change values. AC, articular cartilage; AF, annulus fibrosus; NP, nucleus pulposus.

As the focus of this study was to identify either NP-specific or IVD-specific genes, two separate comparisons were performed. NP cell expression levels were compared with the average expression levels of AC and AF cells (NP/AC&AF) to identify potential NP-specific genes (Table 5), whereas NP cell expression levels and AC expression levels (NP/AC) were compared, without inclusion of AF expression levels, to identify potential IVD-specific genes (Table 6). For both comparisons, stringent thresholds were used to identify differentially expressed genes using a combination of statistical score, expression level and fold change. Statistical significance of differentially expressed genes was assessed with the PUMA Bayesian method [28]. Probesets with a probability of positive log-ratio (PPLR) value less than 0.99 or greater than 0.01 [35] were excluded, as were differentially expressed genes with signal fluorescence intensities less than 50. Although PUMA does not provide false discovery rate (fdr) correction, these thresholds passed by ± 3,000 probesets, correspond approximately to q values of 0.2 if calculated using a parametric ANOVA and fdr correction by qvalue [29]. The results from the NP/AC&AF comparisons identified 185 probesets (127 genes) that had expression intensities greater than 50 in NP cells or in AC&AF cells, were differentially expressed (either up or down regulated) by 10 fold or more and had a PPLR value greater than or equal to 0.99 or less than or equal to 0.01. A subset of 34 of these genes was differentially expressed at least 100 fold (Table 5). The results from the NP/AC comparisons identified 306 probesets (242 genes) that had expression intensities greater than 50, were differentially expressed (either up or down regulated) by 10 fold or more and had a PPLR value greater than or equal to 0.99 or less than or equal to 0.01. A subset of 49 of these genes was differentially expressed by at least 100 fold (Table 6).

**Table 5:** NP vs AC and AF microarray comparison

Gene description	Gene symbol	AC mean	AF mean	AC&AF mean	NP mean	Fold change (NP/AC&AF)
***keratin 8***	***KRT8***	***0.09***	***0.39***	***0.28***	***714.79***	***2544.79***
***keratin 18***	***KRT18***	***0.01***	***0.04***	***0.05***	***94.10***	***1860.77***
ras homolog gene family, member B	RHOB	0.24	0.04	0.40	383.01	**968.68**
hypothetical protein BC012029	LOC152573	0.05	0.18	0.26	230.02	**897.02**
**cadherin 2, type 1, N-cadherin**	**CDH2**	**0.09**	**0.22**	**0.21**	**180.47**	**840.69**
Kruppel-like factor 6	KLF6	0.13	0.03	0.12	55.84	**456.37**
plakophilin 2	PKP2	0.13	0.48	0.37	77.71	**212.23**
related RAS viral (r-ras) oncogene homolog	RRAS	0.74	0.39	0.61	93.40	**152.49**
**synaptosomal-associated protein, 25 kDa**	**SNAP25**	**0.55**	**0.81**	**0.69**	**94.22**	**136.76**
**sclerostin domain containing 1**	**SOSTDC1**	**0.28**	**0.53**	**0.68**	**79.27**	**116.29**
optineurin	OPTN	0.22	0.36	0.80	88.35	**109.86**
*tropomyosin 2 (beta)*	*TPM2*	*18.99*	*94.17*	*78.02*	*0.74*	***-104.73***
integrin, alpha 9	ITGA9	38.74	62.19	66.25	0.50	**-131.50**
*pleiotrophin (heparin binding growth factor 8)*	*PTN*	*208.08*	*54.11*	*167.82*	*1.10*	***-152.58***
Tissue factor pathway inhibitor 2	TFPI2	1080.55	5089.17	4138.76	26.91	**-153.83**
guanine nucleotide binding protein (G protein), gamma 11	GNG11	0.57	331.31	298.19	1.65	**-181.06**
podocalyxin-like	PODXL	0.50	297.24	208.74	1.14	**-182.62**
proteoglycan 4	PRG4	917.06	27.09	722.28	3.25	**-222.06**
a disintegrin-like and metallopeptidase with thrombospondin type 1 motif, 4	ADAMTS4	115.72	2187.60	1561.34	5.25	**-297.16**
phenylalanine hydroxylase	PAH	195.83	0.24	98.50	0.32	**-307.92**
interferon, gamma-inducible protein 16	IFI16	0.28	61.31	67.55	0.20	**-337.80**
retinoic acid receptor responder 1	RARRES1	974.75	156.02	671.46	1.98	**-338.73**
regulator of G-protein signalling 5	RGS5	5.68	436.91	264.83	0.67	**-393.62**
***integrin-binding sialoprotein ***	***IBSP***	***300.90***	***0.06***	***159.69***	***0.37***	***-433.73***
complement factor H	CFH	1.72	60.74	86.66	0.12	**-729.93**
**fibulin 1**	**FBLN1**	**199.29**	**0.22**	**108.88**	**0.13**	**-826.03**
Serpin peptidase inhibitor, clade F, member 1	SERPINF1	17.15	512.43	281.58	0.34	**-830.27**
Keratocan	KERA	0.05	346.47	341.38	0.38	**-893.66**
*collagen, type X, alpha 1*	*COL10A1*	*1192.84*	*7.19*	*741.87*	*0.79*	*-944.44*
Endomucin	EMCN	0.45	281.94	189.76	0.16	**-1166.81**
secreted frizzled-related protein 2	SFRP2	9.98	153.89	106.73	0.08	**-1391.84**
peptidase inhibitor 15	PI15	478.32	83.74	308.94	0.10	**-3231.26**
myosin, heavy chain 11, smooth muscle	MYH11	1.15	462.12	263.70	0.03	**-7708.90**
chemokine (C-X-C motif) ligand 1	CXCL1	3.14	549.84	492.06	0.02	**-28150.12**

NP specific marker genes. Genes identified by NP vs AC and AF microarray comparison. Mean fluorescence intensity is shown for each cell type (AC, AF and NP) along with the mean combined intensity for AC and AF cells and the calculated fold change values (positive and negative) between NP and AC and AF cells that were greater than 100. Highlighted in bold are genes that were taken forward for qRT-PCR and italicised are genes that were identified in previous studies [23,24]. AC, articular cartilage; AF, annulus fibrosus; NP, nucleus pulposus; qRT-PCR, quantitative real time polymerase chain reaction.

**Table 6:** NP vs AC microarray comparison

Gene description	Gene symbol	AC mean	AF mean	NP mean	Fold change (NP/AC)
**brain abundant, membrane attached signal protein 1**	**BASP1**	**0.02**	**57.19**	**384.21**	**16167.90**
***keratin 18 ***	***KRT18 ***	***0.01***	***0.04***	***94.10***	***15990.45***
**Tenomodulin**	**TNMD**	**0.01**	**241.03**	**102.36**	**15087.56**
**TNF, alpha-induced protein 6**	**TNFAIP6**	**0.01**	**304.98**	**121.88**	**8205.96**
***keratin 8***	***KRT8***	***0.09***	***0.39***	***714.79***	***7607.92***
hypothetical protein BC012029	LOC152573	0.05	0.18	230.02	**4635.12**
**TNF, alpha-induced protein 6**	**TNFAIP6**	**0.19**	**3649.85**	**508.71**	**2648.51**
SH3 domain binding glutamic acid-rich protein	SH3BGR	0.08	20.83	172.70	**2230.52**
**cadherin 2, type 1, N-cadherin (neuronal)**	**CDH2**	**0.09**	**0.22**	**180.47**	**1941.17**
chordin	CHRD	0.03	1.29	57.29	1671.45
Rat sarcoma (ras) homolog gene family, member B	RHOB	0.24	0.04	383.01	**1627.81**
homeobox B8	HOXB8	0.08	9.11	119.75	**1591.12**
Rho GTPase activating protein 27	ARHGAP27	0.08	0.72	89.24	**1072.84**
**forkhead box F1**	**FOXF1**	**0.43**	**703.54**	**457.76**	**1054.86**
plakophilin 2	PKP2	0.13	0.48	77.71	**603.25**
homeobox B6	HOXB6	0.11	58.21	61.73	**580.12**
adaptor-related protein complex 2, mu 1 subunit	AP2M1	0.55	350.92	305.70	**557.26**
transketolase-like 1	TKTL1	0.89	4.59	478.18	**536.27**
cytochrome b-245, alpha polypeptide	CYBA	0.16	0.77	83.63	**513.67**
phosphatidylethanolamine-binding protein 4	PEBP4	0.84	113.44	429.31	**511.40**
**forkhead box F2**	**FOXF2**	**0.60**	**416.30**	**283.89**	**470.91**
Kruppel-like factor 6	KLF6	0.13	0.03	55.84	**444.91**
optineurin	OPTN	0.22	0.36	88.35	**407.25**
**aquaporin 1 (Colton blood group)**	**AQP1**	**0.42**	**5.55**	**144.13**	**346.12**
RAB3B, member RAS oncogene family	RAB3B	0.18	0.27	58.47	**333.33**
similar to sushi domain containing 2	SUSD2	1.05	34.26	300.30	**286.96**
**sclerostin domain containing 1**	**SOSTDC1**	**0.28**	**0.53**	**79.27**	**286.23**
CD36 molecule (thrombospondin receptor)	CD36	0.27	96.74	71.61	**262.65**
capping protein (actin filament), gelsolin-like	CAPG	0.21	12.70	53.11	**257.62**
similar to zinc finger homeodomain 4	ZFHX4	0.23	160.03	55.13	**241.22**
neurotrophic tyrosine kinase, receptor, type 2	NTRK2	0.62	248.28	106.60	**172.40**
**synaptosomal-associated protein, 25 kDa**	**SNAP25**	**0.55**	**0.81**	**94.22**	**171.81**
lectin, galactoside-binding, soluble, 1 (galectin 1)	LGALS1	0.57	81.20	88.20	**155.33**
testis derived transcript (3 Lin11, Isl-1 & Mec-3 (LIM) domains)	TES	0.38	32.63	55.66	**145.10**
Related-rat sarcoma viral (r-ras) oncogene homolog	RRAS	0.74	0.39	93.40	**125.71**
sorting nexin family member 30	SNX30	0.74	31.31	89.00	**120.18**
vanin 1	VNN1	0.69	67.28	82.51	**119.73**
collagen, type XVIII, alpha 1	COL18A1	1.02	102.46	116.62	**114.48**
transmembrane protein 100	TMEM100	1.05	1.80	116.48	**110.68**
macrophage migration inhibitory factor	MIF	3.47	1.70	368.05	**105.96**
ectodermal-neural cortex	ENC1	0.74	53.63	75.21	**101.74**
*pleiotrophin*	*PTN*	*208.08*	*54.11*	*1.10*	***-189.18***
cytokine-like 1	CYTL1	11388.79	19.33	29.48	**-386.29**
retinoic acid receptor responder 1	RARRES1	974.75	156.02	1.98	**-491.73**
phenylalanine hydroxylase	PAH	195.83	0.24	0.32	**-612.20**
***integrin-binding sialoprotein ***	***IBSP***	***300.90***	***0.06***	***0.37***	***-817.30***
**fibulin 1**	**FBLN1**	**199.29**	**0.22**	**0.13**	**-1511.93**
*collagen, type X, alpha 1*	*COL10A1*	*1192.84*	*7.19*	*0.79*	***-1518.56***
peptidase inhibitor 15	PI15	478.32	83.74	0.10	**-5002.83**

IVD specific marker genes. Genes identified by NP vs AC microarray comparison. Mean fluorescence intensity is shown for each cell type (AC, AF and NP) along with calculated fold change values (positive and negative) between NP and AC cells that were greater than 100. Highlighted in bold are genes that were taken forward for qRT-PCR and italicised are genes that were identified in previous array studies [23,24]. AC, articular cartilage; AF, annulus fibrosus; NP, nucleus pulposus; qRT-PCR, quantitative real time polymerase chain reaction.

Highlighted in bold in Tables 5 and 6 are genes that were differentially expressed (either up or down regulated) by 100 fold or more and taken forward for qRT-PCR. Italicised are genes that were identified in previously published microarray studies [23,24]. The NP/AC&AF comparisons (Table 5) showed that KRT8 and KRT18 were the highest differentially expressed genes specifically expressed in the NP cells. These were selected for qRT-PCR analysis along with an additional family member, KRT19, previously described in rat arrays [23] and shown here to also be differentially expressed in the bovine NP (Table 4). N-cadherin (CDH2) was also highly differentially expressed and was selected along with synaptosomal-associated protein, 25 kDa (SNAP25) and sclerostin domain containing 1 (SOSTDC1) as NP-specific markers for further analysis. The additional genes, brain abundant, membrane attached signal protein 1 (BASP1), tenomodulin (TNMD), tumor necrosis factor, alpha-induced protein 6 (TNFAIP6), forkhead box F1 (FOXF1), forkhead box F2 (FOXF2) and aquaporin (AQP1) were included from the NP/AC comparisons (Table 6) because they had high differential expression levels but also had detectable levels of expression in the AF and were therefore of interest as transcriptional markers of an IVD cell. AC (negative NP) markers identified by the microarray comparisons included the known hypertrophic chondrocyte marker collagen, type X, alpha 1 (COL10A1; Table 6). The highest differentially expressed genes that were specifically expressed in the AC cells were integrin-binding sialoprotein (IBSP) and fibulin 1 (FBLN1) and were therefore selected as AC (negative NP) markers for further analysis.

### qRT-PCR verification of cell-specific marker genes in bovine samples

**Figure 2:**
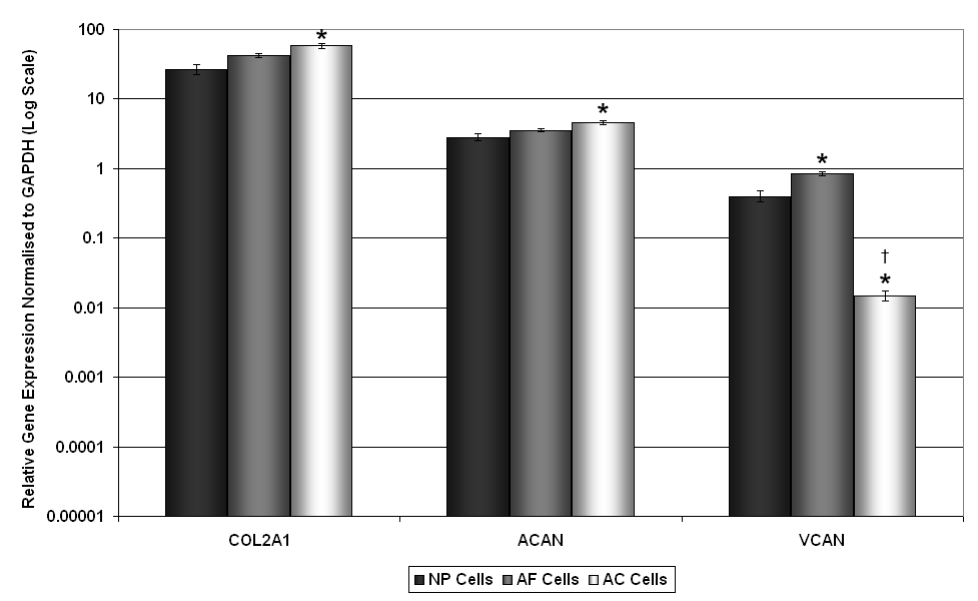
**Quantitative real-time PCR for typical chondrocyte marker genes in bovine AC, AF and NP cells**. Relative gene expression for the chondrocyte marker genes (type II collagen (COL2A1), aggrecan (ACAN) and versican (VCAN)) was normalised to the housekeeping gene, glyceraldehyde-3-phosphate dehydrogenase (GAPDH) and plotted on a log scale. * statistical significance between nucleus pulposus (NP) and annulus fibrosus (AF) cells and nucleus pulposus (NP) and AC cells (*P *< 0.05). † statistical significance between AF cells and AC cells (*P *< 0.05).

qRT-PCR results demonstrated no significant difference in the relative gene expression for the typical chondrocyte marker genes COL2A1 and ACAN between NP and AF or AF and AC cells. However, there were small, but significant, increases in gene expression for COL2A1 and ACAN in AC cells when compared with NP cells (*P *= 0.011 and 0.026, respectively; Figure 2). The expression of VCAN was significantly lower in AC cells when compared with both AF and NP cells (*P *< 0.0001) demonstrating its potential as a gene marker that can distinguish between AC and NP cells.

**Figure 3:**
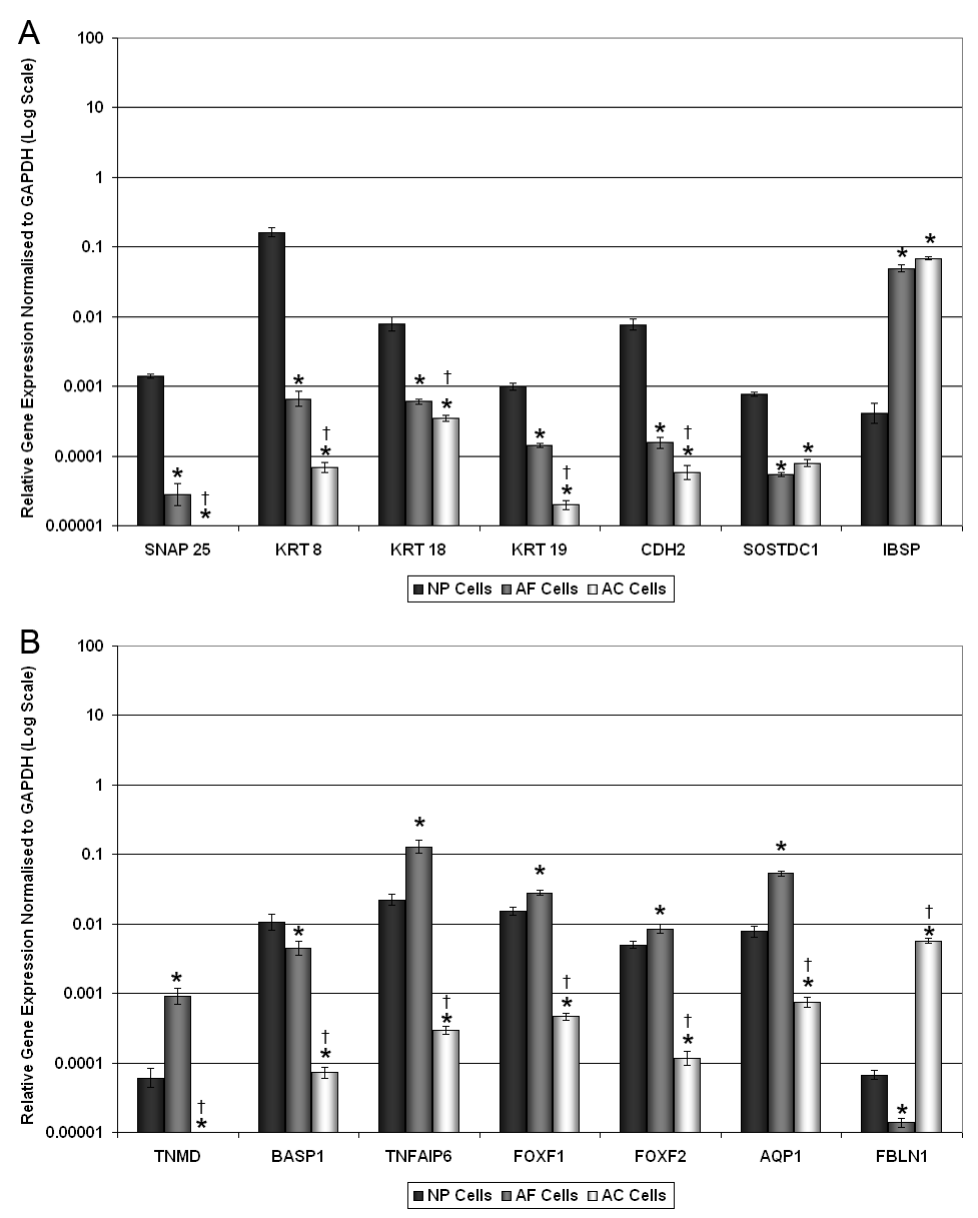
**Quantitative real-time PCR for (a) NP-specific and (b) IVD-specific cell marker genes in bovine AC, AF and NP cells**. Relative gene expression for **(a) **bovine nucleus pulposus (NP)-specific marker genes (synaptosomal-associated protein, 25 kDa (SNAP25), cytokeratin (KRT) 8, KRT18, KRT19, N-cadherin (CDH2), sclerostin domain containing 1 (SOSTDC1) and integrin-binding sialoprotein (IBSP)), and **(b) **intervertebral disc (IVD)-specific cell marker genes (tenomodulin (TNMD), brain abundant, membrane attached signal protein 1 (BASP1), tumor necrosis factor, alpha-induced protein 6 (TNFAIP6), forkhead box F1 (FOXF1), forkhead box F2 (FOXF2), aquaporin (AQP1) and fibulin 1(FBLN1)), was normalised to the housekeeping gene, glyceraldehyde-3-phosphate dehydrogenase (GAPDH) and plotted on a log scale. * statistical significance between NP and annulus fibrosus (AF) cells and NP and articular cartilage (AC) cells (*P *< 0.05). † statistical significance between AF cells and AC cells (*P *< 0.05).

Of the novel NP/IVD marker genes analysed, only SNAP25 and TNMD showed no detectable expression in AC cells after 40 cycles. For SNAP25 (Figure 3a), a very low level of expression was detected in AF cells, which was significantly higher in NP cells (approximately 100 fold, *P *< 0.0001). Conversely, TNMD (Figure 3b) demonstrated a low level of expression in NP cells, which was significantly higher in AF cells (>10 fold, *P *< 0.0001).

Analysis of the NP-specific marker genes (SNAP25, KRT8, KRT18, KRT19, CDH2 and SOSTDC1; Figure 3a) demonstrated significantly higher expression in NP cells when compared with either AF (all genes *P *< 0.0001) or AC cells (all genes *P *< 0.0001), which confirmed the microarray findings. SNAP25, KRT8, KRT18, KRT19 and CDH2 also demonstrated significantly higher expression in AF cells than AC cells (all genes *P *< 0.0001), while SOSTDC1 showed no significant difference between AF and AC cells (*P *= 0.13). The negative NP cell marker gene IBSP demonstrated a similar level of expression in both AF and AC cells, which was significantly higher than that of NP cells (*P *< 0.0001).

Analysis of the IVD-specific marker genes (TNMD, BASP1, TNFAIP6, FOXF1 and FOXF2; Figure 3b) confirmed the microarray results, with expression being significantly higher in both NP and AF cells than AC cells (all genes *P *< 0.0001). In addition, TNMD, TNFAIP6 and AQP1 demonstrated significantly higher expression in AF cells compared with NP cells (all genes *P *< 0.0001), while FOXF1 and FOXF2 demonstrated small, yet significant, increases in AF cells compared with NP cells (*P *< 0.0001). Finally, BASP1, showed a small, but significant increase in NP cells compared with AF cells (*P *= 0.0014). The negative IVD marker gene, FBLN1, showed significantly higher expression in AC cells compared with both NP and AF cells (*P *< 0.0001), with expression in AF cells being significantly lower than in NP cells (*P *< 0.0001).

**Figure 4:**
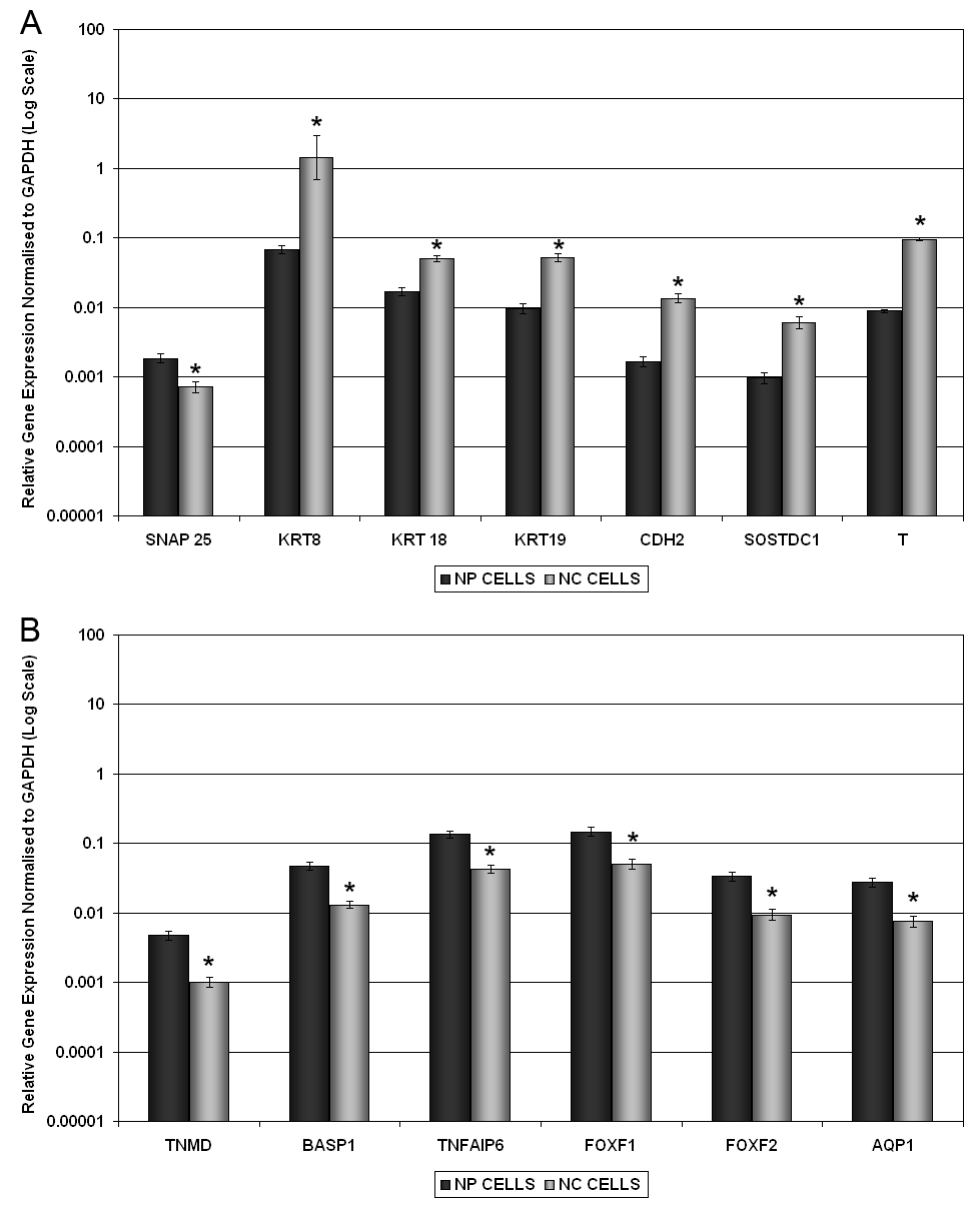
**Quantitative real-time PCR for (a) NP-specific and (b) IVD-specific cell marker genes in separated bovine NP and NC cells**. Relative gene expression for **(a) **bovine nucleus pulposus (NP)-specific marker genes (synaptosomal-associated protein, 25 kDa (SNAP25), cytokeratin (KRT) 8, KRT18, KRT19, N-cadherin (CDH2) and sclerostin domain containing 1 (SOSTDC1)) and the notochord (NC) marker gene (T), and **(b) **intervertebral disc (IVD)-specific cell marker genes (tenomodulin (TNMD), brain abundant, membrane attached signal protein 1 (BASP1), tumor necrosis factor, alpha-induced protein 6 (TNFAIP6), forkhead box F1 (FOXF1), forkhead box F2 (FOXF2) and aquaporin (AQP1)), was normalised to the housekeeping gene, glyceraldehyde-3-phosphate dehydrogenase (GAPDH) and plotted on a log scale. * statistical significance between NP and NC cells (*P *< 0.05).

Although the NP of mature bovine caudal disc is considered to be populated by chondrocyte-like cells, histological assessment of the tissues used here (18 to 36 months old) revealed a small population of resident notochordal cells. To determine whether the identified NP or IVD markers were expressed in either chondrocyte-like NP cells or these larger notochordal cells, the two cell types were isolated and the marker genes detected by qRT-PCR along with the molecular NC marker, brachyury homolog (T) [36,37]. Comparisons of NP (Figure 4a) and IVD (Figure 4b) specific genes in NP cells and NC cells clearly demonstrated expression of each gene by both cell types. Interestingly, NP-specific genes (with the exception of SNAP25) demonstrated significantly higher expression by NC cells than NP cells (all genes *P *< 0.0001), while IVD-specific genes demonstrated significantly higher expression by NP cells than NC cells (all genes *P *< 0.0001). The proposed NC marker gene T, demonstrated expression in both NP and NC cells, but expression was significantly higher in NC cells (*P *< 0.0001).

### qRT-PCR validation of cell-specific marker genes in human samples

#### Gene expression in normal samples

**Figure 5:**
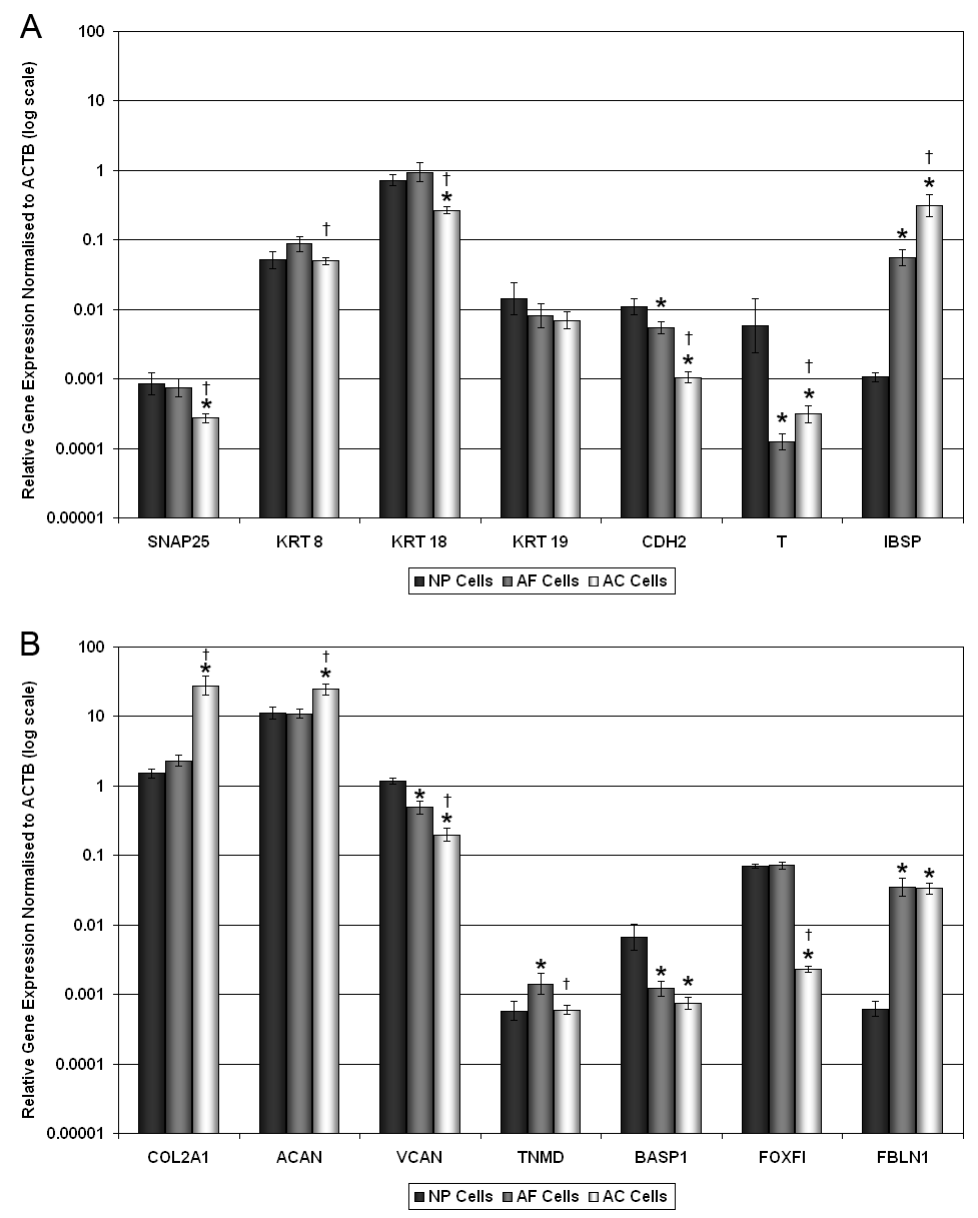
**Quantitative real-time PCR for (a) NP-specific and (b) IVD-specific cell marker genes in normal human AC, AF and NP cells**. Relative gene expression for **(a) **nucleus pulposus (NP)-specific marker genes (synaptosomal-associated protein, 25 kDa (SNAP25), cytokeratin (KRT) 8, KRT18, KRT19, N-cadherin (CDH2) and integrin-binding sialoprotein (IBSP)) and the notochord (NC) marker gene (T), and **(b) **IVD-specific cell marker genes ((tenomodulin (TNMD), brain abundant, membrane attached signal protein 1 (BASP1), forkhead box F1 (FOXF1), and fibulin 1 (FBLN1)) and the chondrogenic marker genes (aggrecan (ACAN) and type II collagen (COL2A1)), was normalised to the housekeeping gene and plotted on a log scale. * statistical significance between NP and annulus fibrosus (AF) cells and NP and articular cartilage (AC) cells (*P *< 0.05). † statistical significance between AF cells and AC cells (*P *< 0.05).

Analysis of expression of traditional marker genes in human normal IVD samples (Figure 5) showed that when compared with AC cells, ACAN and COL2A1 gene expression was significantly lower in normal AF cells (*P *= 0.0003, and *P *< 0.0001, respectively) and normal NP cells (*P *= 0.0018, and *P *< 0.0001, respectively). However, there was no significant difference for either ACAN or COL2A1 between normal AF and NP cells (*P *= 0.39, and *P *= 0.1, respectively).

All novel marker genes analysed were shown to be expressed by human NP, AF and AC cells. In general, a similar pattern of expression was observed for human NP cells as was seen for bovine NP cells. However, differential gene expression levels between the three cell types did not follow a similar trend, with fold changes in gene expression being lower than those observed in bovine cells. In contrast to bovine samples, KRT8, KRT18 and KRT19 showed only small differences in expression between the three cell types studied. KRT8 expression only differed significantly between AF cells and AC cells (*P *= 0.03), while KRT18 expression was significantly higher in NP and AF cells when compared with AC cells (*P *= 0.0006, and *P *< 0.0001, respectively). KRT19 demonstrated no significant differences across any of the cell types. CDH2 showed a similar pattern of expression to bovine samples, with the highest expression in NP cells and the lowest in AC cells. However, although the differences in expression were significant (NP/AF *P *< 0.0001, NP/AC *P *< 0.0001, AF/NP *P *= 0.045), the fold change in expression in human samples was lower than that seen in bovine samples; for example, bovine NP vs AC demonstrated an approximately 100-fold difference, while human NP vs AC an approximately 10-fold difference. The proposed negative NP cell marker IBSP showed significantly lower expression in the NP cells than either the AF or AC cells (*P *< 0.0001), which correlated with the bovine results.

Although the differential expression of TNMD seen in bovine samples between NP and AF cells was preserved in human samples (*P *< 0.05), the fold change was lower (approximately 100-fold in bovine, and approximately 5-fold in human). However, human AC cells showed expression of TNMD, which was absent from bovine AC samples. BASP1 showed a similar pattern of expression in human samples to that demonstrated by bovine cells, although again fold changes were lower between AC and NP cells in human samples than in bovine. Interestingly, in human AF cells the levels of BASP1 expression were closer to those of AC cells than NP cells. FOXF1 showed no difference in expression between NP and AF cells, but both cell types demonstrated significantly higher expression than did AC cells (*P *< 0.0001). In contrast to the bovine samples, the proposed IVD negative marker FBLN1 showed similar levels of expression in both AF and AC cells, but significantly lower expression in NP cells (*P *< 0.0001). In addition, T was expressed in all three human cell types (Figure 5a) with expression significantly higher in NP cells when compared with both AC cells (*P *= 0.02) and AF cells (*P *= 0.004).

### Gene expression in human degenerate IVD samples

**Figure 6:**
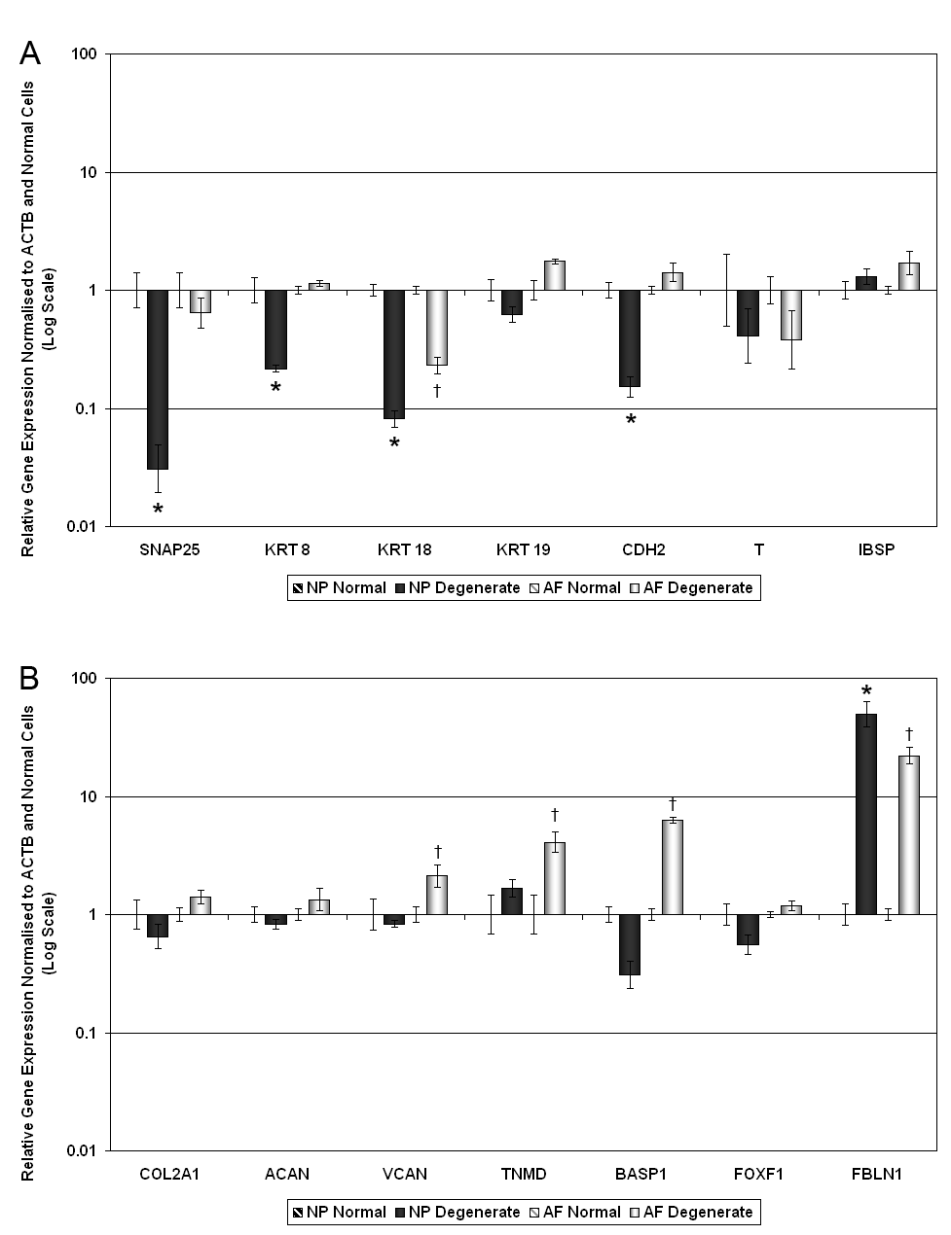
**Quantitative real-time PCR for (a) NP-specific and (b) IVD-specific cell marker genes, in normal and degenerate human AF and NP cells**. Relative gene expression for **(a) **nucleus pulposus (NP)-specific marker genes (synaptosomal-associated protein, 25 kDa (SNAP25), cytokeratin (KRT) 8, KRT18, KRT19, N-cadherin (CDH2) and integrin-binding sialoprotein (IBSP)) and the notochord (NC) marker gene (T), and **(b) **intervertebral disc (IVD)-specific cell marker genes ((tenomodulin (TNMD), brain abundant, membrane attached signal protein 1 (BASP1), forkhead box F1 (FOXF1), and fibulin 1 (FBLN1)) and the chondrogenic marker genes (aggrecan (ACAN) and type II collagen (COL2A1)), was normalised to the housekeeping gene and normal annulus fibrosus (AF) or NP cells and plotted on a log scale. For each gene, expression in normal NP cells or AF cells was plotted on the baseline (value = 1 +/-standard error) and the relative expression in NP or AF degenerate cells (normalised to the relevant normal cell value) was plotted adjacently. * statistical significance between normal and degenerate NP cells (*P *< 0.05). † statistical significance between normal and degenerate AF cells (*P *< 0.05).

For analysis of qRT-PCR data in human samples, the comparative (2^-ΔΔCt^) method was used to demonstrate differences in gene expression between normal and degenerate cells (Figure 6). No significant differences in expression for ACAN or COL2A1 were observed in degenerate samples when compared with normal samples. Analysis of NP-specific marker genes in degenerate samples (Figure 6a) showed a significant decrease in expression for SNAP25, KRT8, KRT18 and CDH2 in degenerate NP cells when compared with normal NP cells (*P *= 0.0002, *P *= 0.0003, *P *< 0.0001 and *P *= 0.0001, respectively). Only KRT18 was significantly decreased in degenerate AF cells (*P *< 0.0001), while KRT19 expression did not differ significantly in degenerate NP or AF cells. For the IVD-specific marker genes TNMD and BASP1 (Figure 6b) there were significant increases in degenerate AF cells when compared with normal AF cells (*P *= 0.03, and *P *< 0.0001, respectively). Interestingly, the negative IVD marker gene (potentially an AC marker) FBLN1 showed significant increases in expression in both degenerate AF cells (approximately 30 fold, *P *< 0.0001) and NP cells (approximately 70 fold, *P *< 0.0001) when compared with normal cells.

## Discussion

NP cells of the IVD share a common lineage with articular chondrocytes, with both cell types expressing the key chondrocyte genes collagen, type II, alpha 1 (COL2A1), aggrecan (ACAN) and SRY (sex determining region Y)-box 9 (SOX-9) [11]. However, the distinctive function of the two tissues is determined by the exact composition of the matrices synthesised by their native cells. Evidence for this comes from a study demonstrating that when chondrocytes from elastic cartilage of rabbit ear were transplanted into the NP of rabbit IVD the ECM formed was predominantly hyaline cartilage, a tissue more solid than the normal NP, which did not function adequately [38]. This implies that for the proper functioning of a tissue engineered IVD it is essential for the implanted cells to have the correct phenotype in order to manufacture a suitable ECM, that is one that resembles the NP and not AC phenotype. However, to date the exact phenotype of a NP cell is not known, although two studies have attempted to elucidate differences in transcriptional profiles between these two cell types in rats and dogs [23,24]. Here, we have used Affymetrix microarrays and qRT-PCR to identify genes that could specifically distinguish bovine NP cells from articular chondrocytes. Microarray results identified a number of genes that were differentially expressed between NP or IVD cells and AC cells. A small number of these genes were then validated by qRT-PCR, which in general confirmed the microarray data, and although no clear on/off marker was identified to distinguish a single cell type it would appear that these novel markers could be used to define a unique transcriptional profile/gene signature that can distinguish NP cells from AC cells in bovines. In addition, analysis in human IVD cells revealed differences in species cell-specific expression, as well as differential expression and alterations with IVD degeneration. Nevertheless, sufficient similarities were identified which may also enable the use of these gene markers to distinguish between human AC and NP cells.

Microarray analysis revealed that typical chondrocyte markers were expressed in all three bovine cell types studied, but that expression levels did not differ sufficiently to distinguish NP cells from AC cells. Interestingly, however, VCAN was expressed significantly higher in AF and NP cells when compared with AC cells. This 87-fold difference in VCAN expression between NP and AC compared with the small fold differences in the other typical marker genes highlights the potential of VCAN as a good marker for differentiating these cell types and corroborates previous literature comparing VCAN content of AC and IVD tissues [39].

The transcriptional profile of phenotypic markers (MMP-2, HIF1A, GLUT-1 and VEGF) identified from studies investigating the unique IVD environment [19-21] did not appear to change significantly in bovine NP cells when compared with AC cells. However, these markers were originally identified from changes in protein expression and it is possible that their differential expression is regulated at the protein level and therefore differs to their transcriptional profiles. Similarly, CD24 [22] was not found to be a useful transcriptional NP marker for bovine cells because it was expressed at a higher level in AC cells than either NP or AF cells. With the exception of KRT18, KRT19 and PTN, the genes identified in rat studies (GPC3, ANXA3, VIM, COMP and MGP) and canine studies (A2M, ANXA4, DSC2, NCAM1) did not show a substantial differential expression in bovine NP cells compared with AC&AF cells. Interestingly, PTN showed substantially higher expression in AC&AF cells when compared with NP cells (approximately 150 fold), contradicting the studies carried out in the rat [23] where it was described as a potential NP marker gene. Such data highlight both the unsuitability of these genes as bovine NP cell markers and the considerable differences that can be observed between the same cell types in different species.

Due to the scale and complexity of cDNA microarray technology it is inherently susceptible to both false-positive and false-negative results. Consequently, microarray data should always be interpreted with caution, especially in the absence of corroborating qRT-PCR data. Two of the genes in this study (ACAN and AQP1) highlight this problem whereby the expression of these genes in AF cells was detected at low levels in the microarray data, while the qRT-PCR data showed expression of these genes at high levels in AF cells. This is most likely due to insufficient hybridisation of the cDNA to that particular array element or errors in detection of the fluorescent signal of the array element. Importantly, such data highlight the necessity to confirm microarray results with qRT-PCR data, which is more reliable and quantitatively accurate. For this reason, genes of interest were further verified using qRT-PCR.

Following comparison of NP/AF&AC cell gene expression from bovine microarrays, six potential NP marker genes were identified and validated by qRT-PCR. KRT8 and KRT18 demonstrated the highest differential expression following microarray analysis, while KRT19 was selected based on a previous rat array study [23], which proposed it as a potential NP marker gene. Although below the stringent threshold (100-fold) used in this study, KRT19 showed significantly higher expression in NP cells when compared with both AC and AF cells (approximately 20-fold). KRT18 is a type I cytokeratin, while KRT8 is a type II keratin that typically dimerises with keratin 18 to form intermediate filaments in the cytoplasm of cells. KRT19 is also a type I cytokeratin but unlike its related family members is not paired with a basic cytokeratin [40]. Members of the cytokeratin family are typically expressed by epithelial cells but are found in a wide range of tissues [41], including the developing NC [42]. Additionally, they are known to be expressed in tissues that are exposed to a fluid or semi-fluid environment [43], suggesting a possible physiological role in ECM homeostasis in the NP.

Our analysis of NP-specific genes also identified for the first time three novel genes (SNAP25, CDH2 and SOSTDC1) not previously reported in the IVD. All three of these genes demonstrated the highest expression in NP cells compared with either AF or AC cells suggesting they may have a specific physiological function in the NP and thus may be useful markers of cell type. Importantly, SNAP25 demonstrated no expression in bovine AC cells and high differential expression between NP and AF cells. The role SNAP25 may play in the NP is unknown, although it is known to be involved in membrane fusion and exocytosis in other tissues [44,45]. Although CDH2 has been previously shown to be important during mesenchymal condensation and chondrogenesis [46,47], the reason for its expression in mature chondrocytes and IVD cells is unclear. SOSTDC1 has been shown to regulate BMP-7 signalling in the kidney [48,49]. As BMP-7 has been shown to prevent NP cell apoptosis [50] and stimulation with recombinant BMP-7 is known to enhance PG production by NP cells [51], SOSTDC1 may play an important role in regulating ECM homeostasis and cell number within the IVD.

Following comparison of NP/AC cell gene expression from bovine microarrays, six potential IVD marker genes were identified, which were validated by qRT-PCR. Analysis confirmed that all six genes (TNMD, BASP1, TNFAIP6, FOXF1, FOXF2 and AQP1) showed significantly higher expression in NP and AF cells than in AC cells. Only BASP1 showed higher expression in NP cells than AF, which has been shown to be expressed in a number of tissues [52-55], although its role within cartilaginous tissues is completely unknown. Importantly, only one of these genes (TNMD) showed no expression in AC cells and given the large differences between its expression in NP and AF cells, TNMD may serve as a marker for the AF cell phenotype. TNMD, is a type II transmembrane protein that shares similarity in its structural configuration with chondromodulin-1 found in chondrocytes [56,57]. It is an anti-angiogenic molecule [58] that is predominantly expressed in dense connective tissues such as tendons and ligaments [57]. Its high expression in the AF, which is a ligamentous tissue, may thus act to inhibit vascular ingrowth into this normally avascular tissue. TNFAIP6 (also known as TNFα-stimulated gene product-6), a molecule expressed in response to inflammatory mediators, has previously been identified in IVD cells where it has been proposed to be involved in ECM homeostasis [59]. Interestingly, the study also highlighted that TNFAIP6 expression in CEPs, a tissue similar to AC, was lower than that seen in either NP or AF cells. Our expression data showed lower expression in AC cells than either NP or AF cells, thus supporting these earlier findings. The genes FOXF1 and FOXF2 showed similar patterns and levels of expression in both NP and AF cells, with lower expression in AC cells. These genes belong to the forkhead family of transcription factors, which have been shown to be involved in cell growth, proliferation, differentiation and longevity [60-62] However, this is the first study to identify their expression within cells of the IVD and therefore their exact roles are yet to be elucidated. AQP-1, previously shown to be expressed by articular chondrocytes [63] and cells of the human IVD [64], where it is believed to act as a bi-directional transmembrane water transport channel, was shown to be expressed more highly in AF cells and NP cells when compared with AC cells, when assessed using qRT-PCR. In contrast, the microarray data showed AQP1 at lower levels in AF cells compared with the other two cell types, which highlights both the susceptibility of microarrays to false-negative results and the importance of confirming microarray results with qRT-PCR.

This study also identified genes that may serve as NP negative (IBSP) or IVD cell negative (FBLN1) markers, in that microarray data showed higher expression in AC cells compared with either NP or AF cells. qRT-PCR validation of IBSP showed similar levels of expression in AF and AC cells, but significantly lower expression in NP cells, thus confirming its potential as a negative NP cell marker. IBSP has also been identified as a potential AC marker gene in a rat microarray study where it showed higher expression levels in AC cells than NP cells [23] but was not further characterised. Validation of FBLN1 also confirmed its potential as a negative IVD cell marker, because its expression was significantly higher in AC cells than in either NP or AF cells.

The identification of NC cells in bovine NP tissue led us to experimentally determine whether the identified NP and IVD markers were expressed either in the smaller chondrocyte-like NP cells or the larger (>15 μm) NC cell population. The two cell types were isolated as previously described on the basis of size [16,65] and the marker genes detected by qRT-PCR along with the molecular NC marker, T [36,37]. The protein encoded by the T gene is an embryonic nuclear transcription factor that binds to a specific DNA element, the palindromic T-site. It binds through a region in its N-terminus, called the T-box, and effects transcription of genes required for mesoderm formation and differentiation. The protein is localised to notochord-derived cells where it is thought to mediate cartilage development in the developing embryo, and also in chordomas, which are believed to arise from cells derived from the notochord [66]. Expression of T in the larger cell population, together with KRT8, KRT18 and KRT19, previously reported to be expressed by notochordal cells [42], suggests that these larger isolated cells are indeed of notochordal origin. Subsequent expression data showed that the NP and IVD genes were expressed by both cell types, with NP marker genes being more highly expressed in the larger NC cells than NP cells and IVD marker genes being higher in NP cells than NC cells. However, differences in expression of these genes between the two cells types was never greater than approximately 10-fold. It has been suggested that the disappearance of NC cells in the mature NP could be due to the differentiation of NC cells towards a chondrocyte-like NP cell and a recent study in mice lends some compelling evidence that all cell types in the adult NP are derived from the NC [67]. Furthermore direct differentiation of NC cells to NP cells has been recently demonstrated *in vitro *using rabbit cells [65]. Importantly the expression of T, KRT8, KRT18 and KRT19 in the smaller NP cells themselves not only confirms these genes as markers of an NP cell but also suggests that these cells are either directly derived from NC cells or that their molecular phenotype is significantly influenced by them. Interestingly, T was also shown to be differentially expressed in human NP cells when compared with AC and AF cells demonstrating the possibility of a similar origin for human NP cells.

Expression of a subset of the validated bovine markers genes (KRT8, 18, 19 SNAP25, CDH2, TNMD, BASP1, FOXF1, IBSP and FBLN 1) was analysed in human cells. Expression of these genes in NP, AF and AC cells was demonstrated, although levels of differential gene expression were reduced between the different cell types. Furthermore, there were also differences in cell-specific gene expression; notably, SNAP 25 and TNMD were expressed by human AC cells and FBLN 1 was expressed by human AF cells at levels very similar to those observed in AC cells. These notable differences in the expression of the NP and IVD markers between bovine and human samples could be explained by interspecies variation, linked to differences in physical or local environmental influences, which have been previously highlighted between rat and canine samples. For example, in addition to the presence of a small population of notochordal cells in young bovine caudal discs, there are also differences in the nutritional and mechanical conditions, which may influence specific gene expression. Furthermore, it is also important to take into account any age-related differences that exist between the two species. Cells harvested from the bovine caudal discs were from animals between 18 months and 3 years old, whereas cells harvested from human samples were from individuals between 45 and 60 years old. Therefore the profile observed in bovine samples may be more indicative of younger cells. Bovine IVD possess an NP ECM which is more PG-rich (and hence has a higher hydration state) than older, more fibrous (and consequently more dehydrated) human discs [68-70], which may also contribute to differences in gene expression profiles between the two species. As such, the genes identified from the bovine array may be indicative of those genes required to generate a more 'hydrogel'-like ECM, rather than the more fibrous NP tissue found in adult aged human discs. Another important consideration that needs to be taken into account is that although articular chondrocytes were isolated from macroscopically and histologically 'normal' regions of cartilage, the cartilage was obtained from joints of patients with osteoarthritis. It is therefore possible that the human AC gene expression profiles could have been altered compared with non-osteoarthritis individuals. Therefore, further studies using either younger human tissues and/or older bovine tissues would be valuable in providing further insight into the effects of age and species on the expression of these gene markers.

Degenerate human NP showed significant decreases in the proposed NP marker genes compared with normal cells, while only KRT18 showed a significant decrease in degenerate AF cells. The fact that the majority of the NP markers showed lower differential expression in human tissues than bovine NP and further decreases in degenerate NP cells supports the argument that these genes could be more characteristic of a young healthy, hydrated IVD. The IVD marker genes TNMD and BASP1 demonstrated increased expression in degenerate AF cells suggesting that they may be involved in one of the many cellular/tissue events characterising the degenerate IVD. Interestingly, levels of FBLN1 (negative IVD cell marker) were significantly increased in both degenerate NP and AF cells. FBLN1 has been shown to bind to ACAN and VCAN [71] as well as a disintegrin-like and metallopeptidase with thrombospondin type 1 motif, 1 (ADAMTS-1), enhancing its capacity to cleave ACAN [72] and it is therefore a regulator of ADAMTS-1-mediated PG proteolysis. As ADAMTS-1 has been shown to be upregulated in IVD degeneration [73], it is possible that the increased expression in FBLN1 may be responsible for, or as a response to, changes in the ECM composition seen in degenerate IVDs.

## Conclusions

In summary, this study has identified a number of novel genes that characterise the bovine NP and IVD cell phenotypes. Although SNAP25 and TNMD may be good markers of bovine NP cells and IVD cells, respectively, given their lack of expression in AC cells, their expression in human AC cells highlights the problems associated with inter-species variation in identifying a single unique NP or IVD cell marker gene. It may therefore be more beneficial to utilise a panel of marker genes, such as those identified here, to differentiate between the different cell types. In fact, the similarity in expression of the IVD marker FOXF1 and the AC marker IBSP between the two species suggests that there are sufficient similarities to merit such an approach. However, for definitive characterisation of a human NP cell or IVD cell phenotype human microarray studies need to be undertaken, validated and also correlated with protein expression to produce a comprehensive molecular signature that will enable researchers to distinguish an NP cell from its closely related AC cell. Achieving this would significantly advance the realisation of tissue engineering strategies that attempt to differentiate MSCs or other progenitor cells towards an NP phenotype.

## Abbreviations

A2M: α-2-macroglobulin; AC: articular cartilage; ACAN: aggrecan; AF: annulus fibrosus; ANOVA: analysis of variance; ANXA4: annexin A4; AQP1: aquaporin; BASP1: brain abundant: membrane attached signal protein 1; CDH2: N-cadherin; CEP: cartilaginous end plates; COL2A1: type II collagen; COL10A1: collagen type X, alpha 1; COMP: cartilage oligomeric matrix protein; DSC: desmocollin 2; ECM: extracellular matrix; FBLN1: fibulin 1; fdr: false discovery rate; FOXF1: forkhead box F1; FOXF2: forkhead box F2; GLUT1: glucose transporter type 1; GPC: glypican; H&E: hematoxylin and eosin; HIF1: hypoxia inducible factor 1 isoforms; HSPS: high salt precipitation solution; IBSP: integrin-binding sialoprotein; IVD: intervertebral disc; KRT: cytokeratin; LBP: low back pain; MGP: matrix Gla protein; MMP: matrix metalloproteinase; MSCs: mesenchymal stem cells; NC: notochord; NCAM1: neural cell adhesion molecule; NP: nucleus pulposus; PCA: principal component analysis; PG: proteoglycan; PPLR: probability of positive log-ratio; PTN: pleiotrophin; qRT-PCR: quantitative real-time polymerase chain reaction; RIN: RNA integrity number; SNAP25: synaptosomal-associated protein, 25 kDa; SOSTDC1: sclerostin domain containing 1; T: brachyury homolog; TNFAIP6: tumor necrosis factor, alpha-induced protein 6; TNMD: tenomodulin; VCAN: versican; VEGF: vascular endothelial growth factor; VIM: vimentin.

## Competing interests

The authors declare that they have no competing interests.

## Authors' contributions

BMM was responsible for the study design, performed all tissue sample preparation, molecular studies, data analysis, and drafted the manuscript. SMR participated in the study design and coordination, analysis of results and helped to draft the manuscript. LAHZ helped in the design, analysis, interpretation and presentation of the microarray data. AJF participated in its design and coordination and analysis of results. JAH conceived the study, secured funding, participated in its design and coordination, analysis of results and co-wrote the manuscript. All authors read and approved the final manuscript.
